# Time Use and Cognitive Achievement among Adolescents in China: Depression Symptoms as Mediators

**DOI:** 10.3390/jintelligence11050088

**Published:** 2023-05-06

**Authors:** Xiaojie Cao, Xinqiao Liu

**Affiliations:** 1 Graduate School of Education, Peking University, Beijing 100871, China; 2 School of Education, Tianjin University, Tianjin 300350, China

**Keywords:** cognitive achievement, time use, depression symptoms, adolescents, mediation

## Abstract

Everyone’s time is limited, and there is competition between different aspects of time use; this requires comprehensive consideration of the effects of different aspects of time use on cognitive achievement in adolescents. This study uses a dataset of 11,717 students from a nationally representative large-scale survey project conducted in 2013 to 2014 to clarify the relationship between time use (including working on homework, playing sports, surfing the Internet, watching TV, and sleeping) and cognitive achievement among Chinese adolescents, and explores the mediating role of depression symptoms in the relationship between time use and cognitive achievement. The results of the correlation analysis show that the average daily time spent on homework, playing sports, and sleeping is significantly positively correlated with cognitive achievement (*p* < 0.01), while time spent surfing the Internet and watching TV are significantly negatively correlated with cognitive achievement (*p* < 0.01). The results of the mediating effect model show that depression symptoms play a mediating role in the relationship between time use and cognitive achievement among Chinese adolescents. Specifically, time spent playing sports (indirect effect = 0.008, *p* < 0.001) and sleeping (indirect effect = 0.015, *p* < 0.001) have a positive effect on cognitive achievement when using depression symptoms as mediators; time spent on homework (indirect effect = −0.004, *p* < 0.001), surfing the Internet (indirect effect = −0.002, *p* = 0.046), and watching TV (indirect effect = −0.005, *p* < 0.001) have a negative effect on cognitive achievement when using depression symptoms as mediators. This study contributes to the understanding of the relationship between time use and cognitive achievement among Chinese adolescents.

## 1. Introduction

Adolescents’ cognitive achievement not only strengthens individual human capital and future competitiveness in the labor market, but also enhances social innovation capacity and promotes economic growth (Cameron and Heckman 1998; Hunter 1986; McLeod and Kaiser 2004; Singh et al. 2019). It is necessary to clarify the mechanisms that contribute to the improvement of adolescents’ cognitive achievement. A growing number of policymakers and parents worldwide are paying attention to the impact of time use on well-being and mental health among adolescents (Borga 2019; Hunt and McKay 2015; Wight et al. 2009). If adolescents allocate a significant proportion of their discretionary time to positive and purposeful constructive activities, this will be beneficial to their healthy growth (Zill 1995). Some studies have analyzed adolescents’ time use. For instance, 81% of schoolchildren aged 11–17 years worldwide do not meet the World Health Organization’s recommendation of one hour of moderate to vigorous physical activity per day, and in China, the figure is 84.3% (Bull et al. 2020; Guthold et al. 2020). In the United States, about 45% of adolescents spend excessive time in front of electronic screens (Onyeaka et al. 2022). However, everyone’s time is limited, and there is competition between different aspects of time use. Considering the impact of only one aspect of time use on cognitive development may not be comprehensive, and all other aspects of time use need to be considered to understand the role of each aspect of time use on adolescents’ cognitive achievement. Thus, clarifying the relationship between time use and cognitive achievement will facilitate the effective utilization of time for adolescent development, and can provide a reference for targeted public health interventions. In this study, cognitive achievement is defined as a comprehensive measure of logical thinking and problem-solving abilities based on standardized cognition tests. Considering the fixed schedules of Chinese adolescents during school hours, we investigate time use outside of school, including time spent on homework, playing sports, surfing the Internet, watching TV, and sleeping.

It is worth analyzing the impact of time use on cognitive achievement mechanisms, as this may shed light on the frequent negative emotions experienced by adolescents in real life (X. Liu et al. 2019b; Pekrun et al. 2002; Pekrun 2006). Depression symptoms are associated with adolescents’ time use and cognitive achievement, and may serve as mediating factors in explaining the relationship between the two. Unfortunately, depression symptoms among adolescents are becoming increasingly prevalent (Cao and Liu 2022; Gao et al. 2022; X. Liu et al. 2019a), with incidence rates rising from 5% at the start of adolescence to as high as 20% at the end of adolescence (Thapar et al. 2012). According to the World Health Organization, one in seven 10- to 19-year-olds worldwide suffers from mental health problems (WHO 2021). The COVID-19 pandemic has exacerbated this trend, with negative time use, such as decreased physical exercise and increased screen time (Neville et al. 2022), leading to cognitive decline and intensified depression symptoms in adolescents (Y. Liu et al. 2021; Xin et al. 2020). Despite this, there is limited research examining the mediating effect of depression symptoms on the relationship between time use and cognitive achievement, particularly in a Chinese context. This study empirically examines the relationship between time use and cognitive achievement among Chinese adolescents and the underlying mechanisms.

### 1.1. The Relationship between Time Use and Cognitive Achievement

Studies have explored the relationship between different aspects of time use and cognitive achievement. Most of the findings suggest that there is a negative relationship between time spent on the Internet and watching television and cognition. This may be because prolonged exposure to the Internet and television can cause changes in the structure and function of the brain, with potentially detrimental effects on higher-order cognition. Additionally, the variety and transience of stimuli on the Internet and television can easily lead to emotional fluctuations and distraction, resulting in cognitive decline (Kuss and Griffiths 2012; Mills 2014). A large-scale survey of American adolescents has revealed a negative correlation between screen time spent on television and social media and cognitive achievement (Walsh et al. 2020). A national survey of 17,076 American adolescents concluded that excessive screen time was detrimental to cognition, increasing the likelihood of cognitive difficulties, such as attention problems and memory impairment, by almost 1.3 times compared to those who did not overuse screens (Onyeaka et al. 2022). Jürges and Khanam (2021) found that educational activities were beneficial to cognitive achievement, while screen time, including watching TV, playing computer games, and social media use, increased internalization problems in Australian adolescents. Islam et al. (2020) discovered that addictive tendencies toward the Internet and electronic games were negatively associated with academic performance in 1704 Australian children aged 11–17 years, and recommended reducing Internet and gaming time through parental supervision and self-discipline. In addition, Internet use and gaming can also significantly disrupt adolescents’ sleep time (King et al. 2013) and increase the risk of mental disorders and dangerous behaviors (Green et al. 2020; Kliestik et al. 2020; Lăzăroiu et al. 2020; Rikkers et al. 2016).

Previous literature has shown that homework time is an important factor in learning ability (Kumar and Choudhury 2021). Parents and teachers tend to believe that spending time of homework can benefit children’s cognitive development (Cooper et al. 2000). Some research has found a significant positive correlation between these two variables. A study based on 8–12-year-old adolescents in India showed that increasing homework time significantly improved the cognitive achievement of students who were lagging behind, suggesting that increasing homework time is one of the key ways to reduce the quality gap between private and public schools in India (Kumar and Choudhury 2021). Reinforcement theory proposes that continuous stimulation leads to changes in the brain’s cognitive and behavioral responses, increasing the likelihood of that behavior. Homework is an effective means of consolidating what students have learned, and spending time on homework helps to reinforce what they have learned in the classroom, thus having a positive effect on cognitive development (Skinner 1954). This has been demonstrated in studies of adolescents in Ethiopia, Vietnam, and India (Borga 2019). There is also evidence that the positive correlation between the two increases with grade level (Cooper and Valentine 2001; Cooper et al. 2006). However, there is currently no consensus on the effect of homework time on cognition, and some studies have found a negative correlation between the two. Chang et al. (2014) found that time spent on homework was negatively correlated with course outcomes based on a survey of 2342 students, possibly because smarter children require less time to complete their homework.

Most studies suggest that there is a significant positive correlation between sleep time and cognitive development. Adequate sleep time can significantly improve cognitive achievement in adolescents, including performance in tasks related to executive function and multiple cognitive domains (Jones and Harrison 2001; Lo et al. 2016). A meta-analysis of 61 studies based on 71 populations showed that sleep restriction has a significant negative effect on executive function, long-term memory, and attention in cognitive performance (Lowe et al. 2017). A survey of 55 adolescents in the Netherlands found that longer sleep time significantly ameliorated visual spatial processing skills (Dewald-Kaufmann et al. 2013). The results from 8323 American adolescents showed that insufficient sleep (less than 9 h) has lasting negative effects on cognition and brain function (Yang et al. 2022). There are also studies suggesting that the correlation between the two is very weak (Astill et al. 2012) or even negative. For example, some surveys of elderly people in Japan and the Bronx found that respondents with longer sleep time had worse cognitive achievement (Kondo et al. 2021; Schmutte et al. 2007).

Insufficient physical activity time presents a critical challenge for today’s adolescents worldwide. In total, 84.6% of Filipino adolescents do not meet the recommended daily minimum of one hour of physical exercise (Cagas et al. 2022). Only 27% of Thai adolescents achieve the daily threshold of 60 min for moderate-to-vigorous physical activity (Widyastari et al. 2022). Prior literature supports a significant positive association between physical activity time and cognitive achievement. Increasing physical activity time can enhance physical and mental functioning, leading to improved cognition (Hillman et al. 2008; Tomporowski et al. 2008). Children who participate in physical activity have larger basal ganglia and hippocampal brain volumes than inactive children, which is associated with superior cognitive control and memory performance (Chaddock-Heyman et al. 2014). A dearth of physical activity can lead to chronic diseases (such as diabetes and obesity) and mental health problems and impose a substantial economic burden on society (Colditz 1999; Hillman et al. 2008). Some studies have produced inconsistent conclusions, with a review of 58 intervention studies finding no conclusive evidence of the beneficial impact of physical activity time on cognitive achievement (Singh et al. 2019). This was also similar to the findings of a survey of Australian fifth–sixth grade primary school students (Dwyer et al. 1983).

Previous literature also suggests possible gender and grade differences in time use (Lloyd et al. 2008). For instance, a survey of American adolescents showed that 8th graders spent more time watching TV and playing video games than 10th graders, and girls spent less time playing video games than boys (Tang and Patrick 2018). Twenge and Martin (2020) found significant gender differences in digital media use among American and British youth, with females spending more time on the Internet and social media, while males allocated more time to gaming and electronic devices. A study of 237 college students found that younger college students had shorter sleep time, while female college students had poorer sleep quality than males (Tsai and Li 2004). A study in Canada found that there were significant gender differences in weekend time use and that adolescents had less leisure time as their grade level increased (Hilbrecht et al. 2008).

In conclusion, existing research lacks a comprehensive analysis of adolescents’ time use across different aspects. Time is a finite resource, and different aspects of time use compete with each other. A narrow focus on a solitary aspect of time use is not effective, and it is crucial to analyze the impact of multiple aspects on adolescents’ cognitive achievement. According to the characteristics and life trajectories of adolescents, their time use typically includes activities such as homework, sports, surfing the Internet, watching TV, and sleeping. It is therefore imperative to investigate the impacts of these common aspects of time use on cognitive achievement among adolescents and give full consideration to gender and grade differences. This will help parents and teachers to improve their parenting and teaching methods effectively.

### 1.2. The Mediating Role of Depression Symptoms

Previous literature has demonstrated a correlation between time use and cognitive achievement, yet little is known about the underlying mechanism. School achievement and psychological well-being are both crucial for adolescents, and a range of factors affect these outcomes, such as parental support (Moè et al. 2020), teacher enthusiasm (Moe et al. 2021), and also depression symptoms (Moè 2015). The present study focuses mostly on the latter variable by examining time use on the one hand, and cognitive achievement on the other. Depression symptoms may serve as the mediating factor that helps to explain this relationship. Control-value theory recognizes that emotions play a crucial role in cognitive processing and behavior. Positive emotions are often seen as a driving force for learning and development, resulting in improved learning efficiency and academic performance. Conversely, negative emotions have a detrimental effect, hindering behavior, learning outcomes, and personal growth (Pekrun et al. 2002; Pekrun 2006). Depression symptoms are the most common negative emotional expression among adolescents worldwide. The World Health Organization estimates that the burden of depression will exceed all other diseases by 2030 (Thapar et al. 2012). The negative impact of depression symptoms on adolescents is becoming increasingly apparent (Gao et al. 2020; X. Liu et al. 2022b), with the incidence among Chinese adolescents rising significantly to 57% following the outbreak of the COVID-19 pandemic (Chen et al. 2021). Unfortunately, few studies have focused on the mediating role of depression symptoms (Lim and Jeong 2022). A study of 562 Puerto Ricans over the age of 60 found that depression symptoms played a complete mediating role in the relationship between perceived daily discrimination and cognitive function (K. Wang et al. 2022). Relevant conclusions drawn from previous studies in other populations cannot be directly applied to the adolescent population, so it is of significance to examine the mediating effect of depression symptoms while taking into account the unique characteristics of adolescence.

Research into the relationship between depression symptoms and cognition has accumulated a substantial body of literature, with the preponderance of evidence indicating that depression symptoms are significant predictors of cognitive decline (Conradi et al. 2011; Lee et al. 2012; Wagner et al. 2012), with 90% of depression patients experiencing impaired cognition (Hollon et al. 2006). A minority of studies have found little association between the two (Goulabchand et al. 2022; Grant et al. 2001). Studies investigating the impact of time allocation for tasks, such as homework and extracurricular activities, on the relationship between depression symptoms and cognition are relatively limited. A survey of Australian adolescents revealed that those with emotional issues or high levels of psychological distress spent the most time on the Internet or playing games (Rikkers et al. 2016). Neuroscientific evidence suggests that chronic sleep deprivation is a risk factor for the onset and progression of depression (Y.-Q. Wang et al. 2015). A study of 82 young adults found that Facebook use was associated with a decrease in subjective well-being. The more frequently people used Facebook, the lower their moment-to-moment experiences of happiness and life satisfaction tended to be (Kross et al. 2013). A review of adolescent mental health during the COVID-19 pandemic found that spending too much time on the Internet and social media can lead to increased symptoms of depression. This highlights how excessive screen time can be detrimental to the mental health of vulnerable adolescents (Oliveira et al. 2022). Although not all studies have confirmed this finding, a synthesis of 25 reviews reveals that the majority of research interprets the association between social media and mental health as weak or inconsistent (Valkenburg et al. 2022). A study of 79 suburban Florida high school students found that those with depression symptoms spent less time on homework and achieved lower average grades (Field et al. 2001). A significant positive association was observed between homework time and depression symptoms among Chinese primary school students in grades 2–6 (Xiao et al. 2022). However, the above literature only examined the relationship between individual time use and depression symptoms separately. Meanwhile, some individual studies have also explored the relationship between time use, cognition, and depression symptoms in specific domains. A survey of 3724 adolescents found that cognitive function moderated the effect of sleep time on depression symptoms (Zhou et al. 2022). A study of 280 retired individuals showed that self-reported depression symptoms were associated with slow stepping reaction time, a connection that could be explained by underlying cognitive impairment (Kvelde et al. 2010).

### 1.3. Aims and Hypotheses

Given the high prevalence of depression symptoms among adolescents and the supporting studies, depression symptoms may play an important mediating role in the relationship between time use and cognitive development. However, there is a paucity of research that comprehensively covers the impact of different aspects of time use on adolescent cognitive achievement, and even fewer studies have empirically examined the mediating effect of depression symptoms between the two. Furthermore, there is a lack of evidence based on in-depth examination of adolescent populations in China. Additionally, the conclusions of previous studies are often based on small sample surveys, which calls into question the generalizability of the findings. What are the characteristics of time use among Chinese adolescents? Do depression symptoms play a mediating role between time use and cognitive achievement? To address these gaps in the literature, this study aims to clarify the relationship between time use and cognitive achievement among Chinese adolescents and the mediating effect of depression symptoms.

**Figure 1:**
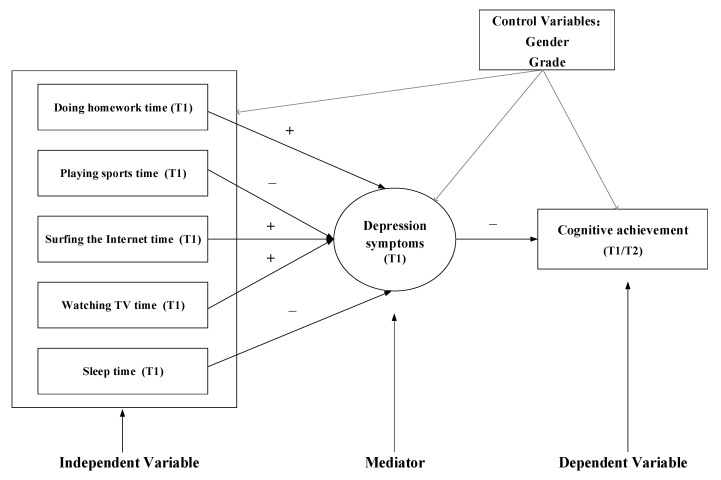
Theoretical research framework. (Note: T1 refers to the 2013–2014 academic year and T2 refers to the 2014–2015 academic year.)

According to the relevant literature and theory, a theoretical research framework was developed (as shown in Figure 1). Based on the literature, the theoretical research framework, and our research objectives, we propose the following research hypotheses:

**Hypothesis 1.**  *There is a significant positive correlation between adolescent average daily time spent on homework, playing sports, and sleeping and cognitive achievement, and a significant negative correlation between average daily time spent surfing the Internet and watching TV and cognitive achievement.*

**Hypothesis 2.**  *Depression symptoms play a mediating role in the relationship between time use and cognitive achievement among adolescents.*

**Hypothesis 2.1.**  *Time spent on homework has a hindering effect on cognitive achievement when using depression symptoms as mediators (more homework time → increases depression symptoms → hinders cognitive achievement).*

**Hypothesis 2.2.**  *Time spent playing sports has a promoting effect on cognitive achievement when using depression symptoms as mediators (more time spent playing sports → decreases depression symptoms → boosts cognitive achievement).*

**Hypothesis 2.3.**  *Time spent surfing the Internet has a hindering effect on cognitive achievement when using depression symptoms as mediators (more time spent surfing the Internet → increases depression symptoms → hinders cognitive achievement).*

**Hypothesis 2.4.**  *Time spent watching TV has a hindering effect on cognitive achievement when using depression symptoms as mediators (more TV time → increases depression symptoms → hinders cognitive achievement).*

**Hypothesis 2.5.**  *Sleep time has a promoting effect on cognitive achievement when using depression symptoms as mediators (more sleep time → decreases depression symptoms → boosts cognitive achievement).*

## 2. Materials and Methods

### 2.1. Participants

The data used in this study were sourced from the 2013–2014 academic year of the China Education Panel Survey (CEPS), which was designed and implemented by the China Survey and Data Center at Renmin University of China. The CEPS is a large-scale, nationally representative survey project. The survey selected 112 schools and 438 classes of seventh and ninth grade students from across the country using a multistage, probability-proportional and scale-based cluster sampling method, stratified by average education level and population proportion. The survey questionnaire was comprehensive, including students’ basic personal information, in-school learning experiences, and extracurricular activities, providing relevant data support for understanding time use, cognition, and depression symptoms among Chinese adolescents. Through data cleaning, a final sample of 11,717 students was retained for empirical analysis. Of these, 5724 were female and 5993 were male, accounting for 48.85% and 51.15% of the sample, respectively; 6161 were in seventh grade and 5556 were in ninth grade, accounting for 52.58% and 47.42% of the sample, respectively.

### 2.2. Measures

Time use: The time use studied in this research specifically involved the following five aspects: time spent on homework, playing sports, surfing the Internet, watching TV, and sleeping. We specifically used the questions “How much on average did you spend on the following extracurricular activities (doing homework time; playing sports time; surfing on the Internet time; watching TV time; sleep time) from Monday to Friday last week?” and “How much time on average did you spend on the following extracurricular activities (doing homework time; playing sports time; surfing on the Internet time; watching TV time; sleep time) last weekend?” from the CEPS student questionnaire. By performing a calculation, we measured the average daily time use (in minutes) of adolescents in the abovementioned aspects. Based on real-life situations, outliers were removed from each variable: time spent on homework, playing sports, surfing the Internet, and watching TV averaged over 300 min per day; sleep time averaged over 720 min per day; and total time averaged over 1440 min per day.

Depression symptoms: We used the CEPS student questionnaire questions “Did you feel blue in the past seven days”, “Did you feel unhappy in the past seven days”, “Did you feel that life was meaningless in the past seven days”, and “Did you feel sad in the past seven days” to measure depression symptom levels. Each item involved a 5-point assessment (ranging from “1 = never” to “5 = always”). Then, we averaged the scores for these four questions, with higher average scores indicating a higher level of depression symptoms in the student. The reliability coefficient α of the depression symptoms scale was 0.8137.

Cognitive achievement: The CEPS designed a cognition test for students that measures their problem-solving ability and logical thinking without involving rote knowledge. The test covers three dimensions, including language, graphics, arithmetic, and logic, with 20 questions in the seventh grade test and 22 questions in the ninth grade test. In our study, we primarily utilized the data obtained from the first round, wherein we employed the number of questions answered correctly by seventh and ninth grade students in a cognitive assessment test to assess their cognitive achievement (T1). The values for this measure ranged from 0 to 20 for the seventh grade cohort and 0 to 22 for the ninth grade cohort. A higher average score on this assessment test indicated a higher level of cognitive achievement among the participants. Moreover, to address the causal inference limitations inherent in cross-sectional data, we incorporated the cognitive achievement (T2) obtained during the second round of data collection as a robustness check for our conclusions.

### 2.3. Data Analysis

First, we utilized Stata to analyze and present descriptive statistical data of the variables of Chinese adolescents’ time use (including working on homework, playing sports, surfing the Internet, watching TV, and sleeping), depression symptoms, and cognitive achievement, as well as gender and grade differences among these variables. Then, we conducted bivariate correlation analysis between the variables of time use, depression symptoms, and cognitive achievement. Finally, we ran Mplus and empirically examined the mediating effect of depression symptoms on the relationship between time use and cognitive achievement through a mediation model.

## 3. Results

### 3.1. Descriptive Statistics

Table 1 presents the mean, standard deviation, minimum, maximum, skewness, and kurtosis of the variables time use, depression symptoms, and cognitive achievement, as well as the gender and grade differences between these variables. The descriptive statistics show that the average score for cognitive achievement (T1) is 10.388 and the average score for depression symptoms is 2.054, indicating normal levels of cognition and depression symptoms. The average daily time spent on homework by Chinese adolescents is 126.401 min, the average daily time spent playing sports is 41.022 min, the average daily time spent surfing the Internet is 34.466 min, the average daily time spent watching TV is 48.139 min, and the average daily sleep time is 472.533 min.

Independent sample *t*-tests reveal that there are significant gender differences in depression symptoms (*t* = 0.067, *p* < 0.01) and time spent on homework (*t* = 14.024, *p* < 0.01), playing sports (*t* = −10.874, *p* < 0.01), surfing the Internet (*t* = −13.918, *p* < 0.01), watching TV (*t* = −4.397, *p* < 0.01), and sleeping (*t* = −5.690, *p* < 0.01), but not in cognitive achievement (T1) (*t* = −0.087, *p* = 0.210). Meanwhile, there are significant grade differences in cognitive achievement (T1) (*t* = 1.818, *p* < 0.01), depression symptoms (*t* = −0.143, *p* < 0.01), and time spent on homework (*t* = −19.640, *p* < 0.01), playing sports (*t* = −1.458, *p* < 0.1), watching TV (*t* = 5.785, *p* < 0.01), and sleeping (*t* = 41.280, *p* < 0.01), but not in time spent surfing the Internet (*t* = −1.250, *p* = 0.148).

**Figure 2:**
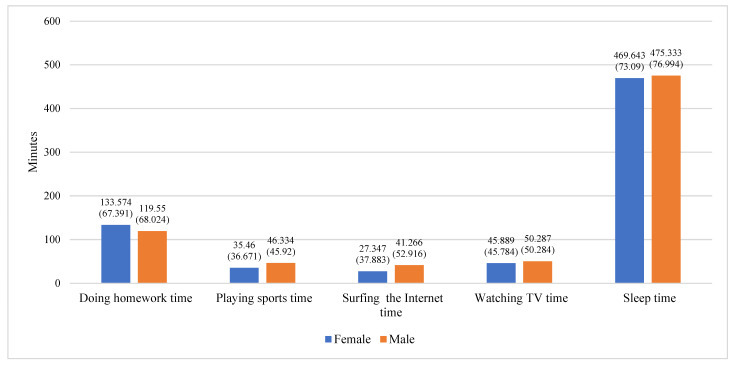
Mean and standard deviation of different aspects of time use by gender (female and male).

**Figure 3:**
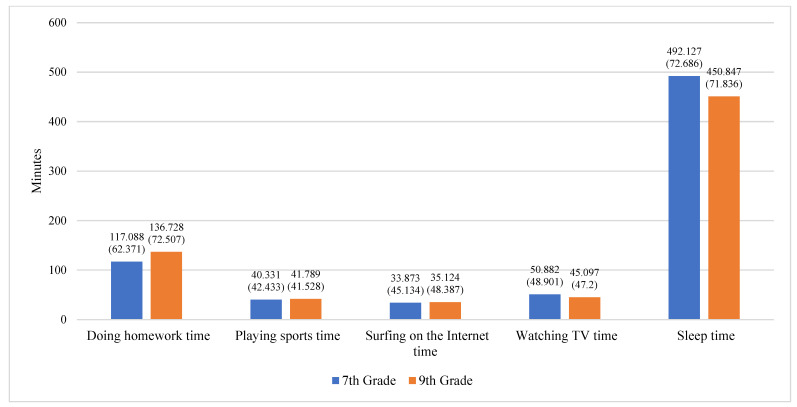
Mean and standard deviation of different aspects of time use by grade (7th grade and 9th grade).

To gain an intuitive understanding of the time use of adolescents of different genders and grades, we also present gender-specific and grade-specific bar charts of different types of time use among Chinese adolescents. As shown in Figure 2, female adolescents (mean = 133.574) spend an average of 14.024 min more per day on homework than male adolescents (mean = 119.55). Female and male adolescents spend, on average, 35.46 min and 46.334 min, respectively, playing sports per day; 27.347 min and 41.266 min, respectively, surfing the Internet per day; 45.889 min and 50.287 min, respectively, watching TV per day; and 469.643 and 475.333 min, respectively, sleeping per day. It can be seen that female adolescents spend less time on average each day playing sports, surfing the Internet, watching TV, and sleeping than male adolescents. As shown in Figure 3, seventh grade and ninth grade students spend an average of 50.882 min and 45.097 min watching TV each day and an average of 492.127 min and 450.847 min sleeping each day, respectively. This demonstrates that seventh grade students spend more time watching TV and sleeping each day than ninth grade students. It is worth noting that ninth grade students (mean = 136.728) spend, on average, almost 20 min more per day on homework than seventh grade students (mean = 117.088). In addition, ninth grade students spend more time on average each day playing sports and surfing the Internet (mean = 41.789; mean = 35.124) than seventh grade students (mean = 40.331; mean = 33.863). In view of these results, we considered gender and grade as control variables in the subsequent analysis.

### 3.2. Correlation Analysis

Table 2 presents the correlation coefficients between time use (time spent on homework time, playing sports, surfing the Internet, watching TV, and sleeping), depression symptoms, and cognitive achievement. The results show that there was a significant negative correlation (*p* < 0.01) between depression symptoms and cognitive achievement (T1 and T2) in Chinese adolescents. There was a significant positive correlation (*p* < 0.01) between the average daily time spent on homework and cognitive achievement (T1 and T2), as well as between time spent on homework and depression symptoms. There was also a significant positive correlation (*p* < 0.01) between time spent playing sports and cognitive achievement (T1 and T2) and a significant negative correlation (*p* < 0.01) between time spent playing sports and depression symptoms. A significant negative correlation (*p* < 0.01) was found between time spent surfing the Internet and cognitive achievement (T1 and T2), and a significant positive correlation (*p* < 0.01) was found between time spent surfing the Internet and depression symptoms. A significant negative correlation (*p* < 0.01) was found between time spent watching TV and cognitive achievement (T1 and T2), and a significant positive correlation (*p* < 0.01) was found between time spent watching TV and depression symptoms. There was a significant positive correlation (*p* < 0.01) between sleep time and cognitive achievement (T1) and a significant negative correlation (*p* < 0.01) between sleep time and depression symptoms. This verifies hypothesis 1. Additionally, aside from the insignificant correlation between time spent playing sports and both surfing the Internet (*p* = 0.496) and watching TV (*p* = 0.993), all other variables exhibit significant interrelationships.

### 3.3. Mediation Model

We controlled for gender and grade and used a mediation model and bootstrap method to examine the mediating effect of depression symptoms between time use and cognitive achievement (T1). The model fit indices are good (RMSEA = 0.063, CFI = 0.914, SRMR = 0.039). Table 3 presents the results of the model. In the mediation model, the direct effect of time spent on homework on cognitive achievement (T1) is 0.138 (*p* < 0.001), with a confidence interval of [0.122, 0.156], indicating a significant direct effect; the indirect effect of depression symptoms between the two is −0.004 (*p* < 0.001), with a confidence interval of [−0.006, −0.002], indicating a significant indirect effect. The direct effect of time spent playing sports on cognitive achievement (T1) is 0.038 (*p* < 0.001), with a confidence interval of [0.019, 0.056], indicating a significant direct effect; the indirect effect of depression symptoms between the two is 0.008 (*p* < 0.001), with a confidence interval of [0.006, 0.011], indicating a significant indirect effect. The direct effect of time spent surfing the Internet on cognitive achievement (T1) is 0.001 (*p* = 0.899), with a confidence interval of [−0.017, 0.019], indicating an insignificant direct effect; the indirect effect of depression symptoms between the two is −0.002 (*p* = 0.046), with a confidence interval of [−0.005, 0.000], indicating a significant indirect effect. The direct effect of time spent watching TV on cognitive achievement (T1) is −0.117 (*p* < 0.001), with a confidence interval of [−0.133, −0.101], indicating a significant direct effect; the indirect effect of depression symptoms between the two is −0.005 (*p* < 0.001), with a confidence interval of [−0.008, −0.003], indicating a significant indirect effect. The direct effect of sleep time on cognitive achievement (T1) is −0.013 (*p* = 0.202), with a confidence interval of [−0.032, 0.006], indicating an insignificant direct effect; the indirect effect of depression symptoms between the two is 0.015 (*p* < 0.001), with a confidence interval of [0.011, 0.019], indicating a significant indirect effect. In addition, both gender (*p* = 0.025) and grade level (*p* < 0.001) significantly affect cognitive achievement (T1).

The above results indicate that depression symptoms play a mediating role in the relationship between time use and cognitive achievement (T1) among Chinese adolescents, supporting Hypothesis 2. Specifically, time spent playing sports and sleeping have a positive effect on cognitive achievement (T1) when using depression symptoms as mediators; time spent on homework, surfing the Internet, and watching TV have a negative effect on cognitive achievement (T1) when using depression symptoms as mediators.

To further address the challenge of making causal inferences with cross-sectional data (Baiden et al. 2019), we conducted a robustness check on our research findings using the data from the second wave of cognitive achievement tests. The purpose of this was to demonstrate that our current results are still valid in longitudinal data, as shown in Table 4. By comparing the results from Table 3 and Table 4, we observe that the findings regarding the mediating effect are consistent, thus indicating the reliability of our current results.

## 4. Discussion

This study comprehensively examines the relationship between time use and cognitive achievement among Chinese adolescents in common activities, including working on homework, playing sports, surfing the Internet, watching TV, and sleeping. Importantly, we clarify the mediating effect of depression symptoms between time use and cognitive achievement. Our findings provide new valuable evidence in a Chinese context that adds to the literature.

The results of our descriptive analysis reveal that, excluding time spent sleeping, adolescents in China spend the most time on homework. The insufficient daily physical activity time among Chinese adolescents (less than one hour) also deserves a high level of attention. This aligns with trends found in previous large-scale global surveys based on approximately 150 countries and the latest reports from other countries, such as the Philippines and Thailand (Cagas et al. 2022; Guthold et al. 2020; Widyastari et al. 2022). Given the importance of physical exercise, it is necessary to take measures to motivate adolescents to actively participate in physical exercise as soon as possible, thus contributing to the happiness and healthy growth of adolescents globally. Additionally, our analysis of the gender and grade differences in time use revealed that compared to male adolescents, female adolescents invested more time in homework, and correspondingly, spent less time sleeping, playing sports, surfing the Internet, and watching TV. This not only reflects differences in time utilization preferences between genders, but also highlights the tendency of individuals to allocate their limited time toward what they value most. Furthermore, this trend can also be observed in China, where female students tend to have higher academic engagement, which may be due to the traditional cultural notion of “emphasizing boys over girls” in certain regions. To achieve high grades and gain recognition, female students may have to work harder through diligent study to secure their futures. Additionally, the largest change in time use among Chinese adolescents from grades 7 to 9 is a significant increase in average daily homework time, accompanied by an almost 1 h reduction in sleep time. In China, where exam-oriented Confucian culture is highly revered, adolescents in middle school face intense academic competition. Increasing grade level often translates to a heavier academic load (Fu et al. 2022), especially as the importance of homework becomes more apparent (Cooper and Valentine 2001; Kumar and Choudhury 2021). Thus, a growing number of middle school students in developing countries have to sacrifice sleep time to improve their learning time and boost their competitiveness within the educational system (Lloyd et al. 2008).

The scarcity of time makes people value the positive effects of utilizing it even more. Our correlational analysis found that the average daily time spent doing homework, playing sports, and sleeping were significantly positively correlated with cognitive achievement, while time spent surfing the Internet and watching TV were significantly negatively correlated with cognitive achievement. Specifically, the relationship between homework time and cognitive achievement aligns with previous research (Borga 2019; Cooper and Valentine 2001; Cooper et al. 2006; Kumar and Choudhury 2021) and accommodates the expectations of teachers and parents (Cooper et al. 2000). According to Skinner’s reinforcement theory, the increase in homework time and the continual repetition of content and knowledge leads to strengthened learning and improved cognitive development and academic outcomes (Skinner 1954). It is worth noting that there is also a significant positive correlation between homework time and depression symptoms, which, in turn, has a significant negative correlation with cognitive achievement. The findings of this study are consistent with previous research on the association between homework time and depression symptoms in Chinese primary school students (Xiao et al. 2022), indicating a common phenomenon in China. This relationship may serve as an indicator of stress in students. If they feel the need to spend more time on homework, it may be due to concerns about their future, meeting parental expectations, and worries about their opportunities for higher education in China, where competition for college positions is fierce. Chronic stress of this type can lead to symptoms of depression. Therefore, the relationship between homework time and cognitive achievement needs further investigation, considering depression symptoms as a mediating factor. The correlation between time spent playing sports and cognitive achievement confirms previous research (Hillman et al. 2008; Tomporowski et al. 2008) but contradicts findings from Singh et al. (2019) and Dwyer et al. (1983). The relationship between sleep time and cognitive achievement is consistent with most previous research (Dewald-Kaufmann et al. 2013; Jones and Harrison 2001; Lo et al. 2016; Lowe et al. 2017; Yang et al. 2022), indicating that adequate sleep benefits the cognition of adolescents. This differs from the conclusions of Kondo et al. (2021) and Schmutte et al. (2007), which may be due to their focus on older individuals. This suggests that the effects of time use on cognitive achievement may vary among different groups and deserves attention from future researchers. In addition, the conclusions regarding the relationship between time spent surfing the Internet and watching TV and cognitive achievement are consistent with previous studies (Islam et al. 2020; Jürges and Khanam 2021; Onyeaka et al. 2022; Walsh et al. 2020), demonstrating that excessive screen time among Chinese adolescents, as with other countries, can harm cognitive development.

Moreover, this study also confirms the mediating role of depression symptoms between time use and cognitive development in Chinese adolescents. Based on the mediating effect model, and after controlling for gender and grade, we found that different aspects of time use had varying effects on the path of cognitive development. Time spent on homework, playing sports, and watching TV could either directly impact cognitive development or indirectly impact it when using depression symptoms as mediators, whereas time spent surfing the Internet and sleeping only impacts cognitive development when using depression symptoms as mediators. Notably, the impact of depression symptoms as mediating variables on cognitive development varies between different time utilizations. Working on homework, surfing the Internet, and watching TV had a negative impact on cognitive development when using depression symptoms as mediators, while playing sports and sleeping had a positive impact on cognitive development when using depression symptoms as mediators. These findings, combined with our previous results, suggest that increasing time spent on homework, surfing the Internet, and watching TV increases the level of depression symptoms and, in turn, reduces cognitive achievement. On the other hand, increasing time spent playing sports and sleeping reduces depression symptoms and enhances cognitive achievement. These findings highlight the complexity of the relationship between time use and cognitive achievement in adolescents and, to some extent, support the role of negative emotions in affecting learning behaviors and cognitive performance (Pekrun et al. 2002; Pekrun 2006), as proposed in control-value theory. Thus, the negative effects of adolescent negative emotions should not be ignored (X. Liu et al. 2022a, 2023b; Thapar et al. 2012).

This study has important implications and references for educational practice. First, under the premise that time is limited and different time uses mutually compete, our results reveal that time use pertaining to academics, physical health, and sleep may be more sensible and effective for promoting cognitive achievement among adolescents. It should be noted that the results of our mediating model show that more time spent on homework does not necessarily equate to better cognitive achievement. We found a significant positive correlation between homework time and depression symptoms, a significant negative correlation between depression symptoms and cognitive achievement, and an adverse effect of time spent on homework on cognitive development using depression as a mediator. This suggests that more homework time leads to higher levels of depression and hinders cognitive development. Future studies may consider conducting randomized intervention trials to further explore the most scientific and reasonable academic tasks and completion times that schools should set. This may also be an urgent issue that policy makers, educators, and parents in other countries with similar education competition and limited resources to China should understand (Cao et al. 2023; X. Liu et al. 2023a). Second, our findings also suggest that the occurrence of depression symptoms among adolescents may be an important signal and a key mediator connecting adolescent behavior and cognition. This also makes up for the lack of attention to the mediating role of depression symptoms in previous literature (Lim and Jeong 2022; K. Wang et al. 2022). Third, in order to enhance the alleviation of depression symptoms among adolescents in educational settings, it is imperative that education management departments, schools, and parents work together to attach great importance to the psychological health development of adolescents worldwide. This can be achieved by fostering a relaxed learning atmosphere through joint efforts between families and schools. It is preferable to identify students at risk of depression as early as possible and to take timely action to improve adolescents’ mental health and cognitive achievement, thereby promoting their comprehensive growth.

## 5. Limitations

First, limited by the database, the correlations between time use, cognitive achievement, and depression symptoms were obtained based on cross-sectional data. In the future, if there are data for tracking relevant variables, further analysis of the longitudinal relationships between variables will be considered. Second, based on the items available in the database, we used four questions to assess the depression symptom levels of adolescents. It would be beneficial to validate these findings using other depression symptoms scales in future studies. Third, due to the limitations of the database, we were unable to obtain specific details of adolescent time use, such as the types of sporting activities they engage in; these deserve further exploration by relevant researchers in the future. Fourth, data on time use, cognitive achievement, and depression symptoms all came from self-reported reports in student questionnaires, so there may be some measurement bias. Fifth, we conducted a mediation analysis on the large-scale survey data using methods similar to those used in previous analyses. Although we considered a complex survey design for estimating standard errors in the analyses, the current data did not support its inclusion. This highlights an area for future research and improvement. Finally, although the conclusions of this study are instructive for parents, teachers, or other groups concerned with related issues, the relatively small effect sizes obtained from such a large sample should be interpreted with caution. Nevertheless, we believe that the evidence is still very valuable.

## 6. Conclusions

First, the average daily time spent on homework, playing sports, and sleeping are significantly positively correlated with cognitive achievement, while the time spent surfing the Internet and watching TV are significantly negatively correlated with cognitive achievement.

Second, time spent playing sports and sleeping have a positive effect on cognitive achievement when using depression symptoms as mediators.

Third, time spent on homework, surfing the Internet, and watching TV have a negative effect on cognitive achievement when using depression symptoms as mediators.

## Figures and Tables

**Table 1:** Descriptive statistics of cognitive achievement, depression symptoms, and time use and their gender and grade differences.

Variables	N	Mean	Standard Deviation	Min	Max	Skewness	Kurtosis	Gender Difference	Grade Difference
Cognitive achievement (T1)	11,717	10.388	3.753	0	22	0.007	2.564	−0.087	1.818 ***
Cognitive achievement (T2)	10,750	21.072	8.969	0	35	−0.915	3.271	0.726 ***	-
Depression symptoms	11,526	2.054	0.794	1	5	0.928	4.227	0.067 ***	−0.143 ***
Time spent on homework	11,717	126.401	68.075	0	300	0.21	2.53	14.024 ***	−19.640 ***
Time spent playing sports	11,717	41.022	42.011	0	300	1.483	6.275	−10.874 ***	−1.458 *
Time spent surfing the Internet	11,717	34.466	46.707	0	300	2.182	8.918	−13.918 ***	−1.250
Time spent watching TV	11,717	48.139	48.187	0	300	1.684	6.676	−4.397 ***	5.785 ***
Sleep time	11,717	472.553	75.163	0	720	−1.192	8.636	−5.690 ***	41.280 ***

Note: *** 1% significance level, * 10% significance level; *T*-tests were used to determine gender and grade differences; the times in the table represent minutes per day. Gender Difference is defined as the difference between the values of the variable for females and males. Grade Difference is defined as the difference between the values of the variable for 7th and 9th graders. T1 refers to the 2013–2014 academic year and T2 refers to the 2014–2015 academic year.

**Table 2:** Correlation analysis of cognitive achievement, depression symptoms, and time use.

Variables	(1)	(2)	(3)	(4)	(5)	(6)	(7)	(8)
(1) Cognitive achievement (T1)	1 -							
(2) Cognitive achievement (T2)	0.326 *** [0.319, 0.357]	1 -						
(3) Depression symptoms	−0.105 *** [−0.124, −0.087]	−0.096 *** [−0.115, −0.077]	1 -					
(4) Time spent on homework	0.108 *** [0.090,0.127]	0.105 *** [0.086, 0.124]	0.055 *** [0.037, 0.073]	1 -				
(5) Time spent playing sports	0.048 *** [0.030,0.066]	0.047 *** [0.028, 0.066]	−0.084 *** [−0.102, −0.066]	0.028 *** [0.010, 0.046]	1 -			
(6) Time spent surfing the Internet	−0.044 *** [−0.062,−0.026]	−0.108 *** [−0.127, −0.09]	0.045 *** [0.027, 0.063]	−0.115 *** [−0.134, −0.097]	0.006 [−0.012, 0.024]	1 -		
(7) Time spent watching TV	−0.120 *** [−0.139,−0.102]	−0.124 *** [−0.144, −0.106]	0.053 *** [0.035, 0.071]	−0.109 *** [−0.128, −0.091]	0.000 [−0.018, 0.018]	0.226 *** [0.212, 0.248]	1 -	
(8) Sleep time	0.043 *** [0.025,0.061]	0.014 [−0.005, 0.033]	−0.157 *** [−0.177, −0.140]	−0.216 *** [−0.238, −0.201]	−0.009 [−0.027, 0.009]	−0.055 *** [−0.073,−0.037]	0.024 ** [0.006, 0.042]	1 -

Note: *** 1% significance level, ** 5% significance level. The 95% confidence interval calculated using the Fisher transformation method for the correlation coefficient is presented below; T1 refers to the 2013–2014 academic year and T2 refers to the 2014–2015 academic year.

**Table 3:** Mediating effect of depression symptoms on the relationship between time use and cognitive achievement (T1) among Chinese adolescents.

Pathways	Estimate	95% CI		S.E.	Estimate/S.E.	*p* Value
		Lower	Upper			
**Direct effects**						
Time spent on homework → cognitive achievement (T1)	0.138	0.122	0.156	0.009	15.981	0.000
Time spent playing sports → cognitive achievement (T1)	0.038	0.019	0.056	0.009	3.980	0.000
Time spent surfing the Internet → cognitive achievement (T1)	0.001	−0.017	0.019	0.009	0.127	0.899
Time spent watching TV → cognitive achievement (T1)	−0.117	−0.133	−0.101	0.008	−14.314	0.000
Sleep time → cognitive achievement (T1)	−0.013	−0.032	0.006	0.010	−1.276	0.202
**Indirect effects**						
Time spent on homework → depression symptoms → cognitive achievement (T1)	−0.004	−0.006	−0.002	0.001	−3.686	0.000
Time spent playing sports → depression symptoms → cognitive achievement (T1)	0.008	0.006	0.011	0.001	6.230	0.000
Time spent surfing the Internet → depression symptoms → cognitive achievement (T1)	−0.002	−0.005	0.000	0.001	−1.997	0.046
Time spent watching TV → depression symptoms → cognitive achievement (T1)	−0.005	−0.008	−0.003	0.001	−4.781	0.000
Sleep time → depression symptoms → cognitive achievement (T1)	0.015	0.011	0.019	0.002	7.585	0.000

Note: T1 refers to the 2013–2014 academic year and T2 refers to the 2014–2015 academic year.

**Table 4:** Indirect effect of depression symptoms on the relationship between time use and cognitive achievement (T2) among Chinese adolescents.

Pathways	Estimate	95% CI		S.E.	Estimate/S.E.	*p* Value
		Lower	Upper			
Time spent on homework → depression symptoms → cognitive achievement (T2)	−0.004	−0.007	−0.002	0.001	−3.322	0.001
Time spent playing sports → depression symptoms → cognitive achievement (T2)	0.009	0.005	0.012	0.002	4.975	0.000
Time spent surfing the Internet → depression symptoms → cognitive achievement (T2)	−0.002	−0.005	0.000	0.001	−1.923	0.054
Time spent watching TV → depression symptoms → cognitive achievement (T2)	−0.005	−0.009	−0.003	0.001	−4.316	0.000
Sleep time → depression symptoms → cognitive achievement (T2)	0.015	0.010	0.021	0.003	5.524	0.000

Note: T1 refers to the 2013–2014 academic year and T2 refers to the 2014–2015 academic year.

## Data Availability

The data used in this study were obtained from the following website: http://ceps.ruc.edu.cn/ (accessed on 7 January 2023).
